# Tele-EMS physicians improve life-threatening conditions during prehospital emergency missions

**DOI:** 10.1038/s41598-021-93287-5

**Published:** 2021-07-13

**Authors:** Hanna Schröder, Stefan K. Beckers, Klaudia Ogrodzki, Christina Borgs, Sebastian Ziemann, Andreas Follmann, Rolf Rossaint, Marc Felzen

**Affiliations:** 1 Department of Anesthesiology, Medical Faculty RWTH Aachen University, University Hospital RWTH Aachen, Pauwelsstrasse 30, 52074 Aachen, Germany; 2 Aachen Institute for Rescue Management & Public Safety, City of Aachen and University Hospital RWTH Aachen, Pauwelsstrasse 30, 52074 Aachen, Germany; 3 Medical Direction of Aachen Fire Department, Stolbergerstrasse 155, 52068 Aachen, Germany; 4 Dental Practice of Dr. Marc Schmidt, Zähringerplatz 7, 78464 Konstanz, Germany

**Keywords:** Health care, Medical research, Signs and symptoms

## Abstract

Almost seven years ago, a telemedicine system was established as an additional component of the city of Aachen’s emergency medical service (EMS). It allows paramedics to engage in an immediate consultation with an EMS physician at any time. The system is not meant to replace the EMS physician on the scene during life-threatening emergencies. The aim of this study was to analyze teleconsultations during life-threatening missions and evaluate whether they improve patient care. Telemedical EMS (tele-EMS) physician consultations that occurred over the course of four years were evaluated. Missions were classified as involving potentially life-threatening conditions based on at least one of the following criteria: documented patient severity score, life-threatening vital signs, the judgement of the onsite EMS physician involved in the mission, or definite life-threatening diagnoses. The proportion of vital signs indicating that the patient was in a life-threatening condition was analyzed as the primary outcome at the start and end of the tele-EMS consultation. The secondary outcome parameters were the administered drug doses, tracer diagnoses made by the onsite EMS physicians during the missions, and quality of the documentation of the missions. From January 2015 to December 2018, a total of 10,362 tele-EMS consultations occurred; in 4,293 (41.4%) of the missions, the patient was initially in a potentially life-threatening condition. Out of those, a total of 3,441 (80.2%) missions were performed without an EMS physician at the scene. Records of 2,007 patients revealed 2,234 life-threatening vital signs of which 1,465 (65.6%) were remedied during the teleconsultation. Significant improvement was detected for oxygen saturation, hypotonia, tachy- and bradycardia, vigilance states, and hypoglycemia. Teleconsultation during missions involving patients with life-threatening conditions can significantly improve those patients' vital signs. Many potentially life-threatening cases could be handled by a tele-EMS physician as they did not require any invasive interventions that needed to be performed by an onsite EMS physician. Diagnoses of myocardial infarction, cardiac pulmonary edema, or malignant dysrhythmias necessitate the presence of onsite EMS physicians. Even during missions involving patients with life-threatening conditions, teleconsultation was feasible and often accessed by the paramedics.

## Introduction

From 2007 to 2013, the Department of Anesthesiology of the University Hospital RWTH Aachen and several partners developed a holistic prehospital telemedicine system intended to supplement the conventional prehospital emergency medical service (EMS). The EMS system in Germany is based on a rendezvous-system between ambulances staffed with paramedics and a vehicle with an emergency physician. The operator in the EMS dispatcher center decides to send either only the ambulance or both vehicles in the case of a potentially life-threatening emergency^1^. The implemented telemedicine system enables every paramedic to contact a telemedical EMS (tele-EMS) physician anytime and anywhere^2–4^. The system was fully implemented as a complement to the regular 24/7 routine EMS in Aachen in April 2014. Since then, the number of telemedicine consultations for prehospital emergency patients continuously increased each year and became part of the daily routine for paramedics. Teleconsultation takes place on a voluntary basis but also follows the existing standard operating procedures (SOPs) for 12 traced diagnoses^5^.

The system has been shown to improve several aspects of prehospital patient care. It reduces the physician-free interval by enabling paramedics to access an immediate consultation. On the one hand, the tele-EMS physician can offer support and delegate the administration of drugs to achieve earlier treatment. Acute pain can be relieved immediately by pain medication prescribed by the tele-EMS physician without impairing the quality of care^6–8^. An increasing use of teleconsultations during prehospital management and the interhospital transfer of stroke patients has been reported and further been shown to facilitate better transfer of specific stroke information and more accurate decision making^9–11^. On the other hand, the tele-EMS physician can bridge the time gap between diagnosis and treatment for patients with life-threatening diseases and injuries until the EMS physician arrives at the scene. Consequently, telemedicine systems ensure a higher quality of emergency medical care at an earlier stage.

Prehospital tele-emergency medicine was not intended to replace onsite EMS physicians in life-threatening emergencies.

In contrast, the major goal was to supplement the EMS resources available for missions during which the skills of a physician are not needed at the scene, but drugs need to be administered or decisions need to be made^5,12^. Nevertheless, teleconsultations are regularly conducted during missions involving patients with potentially life-threatening missions mainly due to the management in the dispatch center. Usually, the national indication index for missions needing EMS physicians onsite, published by the German Medical Association, functions as a guide for dispatch center operators^13^. Nevertheless, the medical director of a regional EMS system may also adapt the indication index to match regional characteristics (e.g., the existence of a telemedicine system) based on his own judgement. It is always the primary intention to dispatch the EMS physician specifically to patients with life-threatening conditions (e.g., myocardial infarction, severe accidents, acute dyspnea) and not to waste this valuable resource on minor events. When an emergency call does not reveal that the patient is in a critical condition, and the emergency presents as minor, the operators only alert the paramedic-staffed ambulance. Upon arriving at the scene, it is the paramedics’ duty to evaluate the situation and decide how best to handle it. In addition to handling the mission themselves, they can call for an onsite EMS physician or conduct a teleconsultation. Consequently, situation do occur when paramedics find that the patient is actually in a life-threatening situation and decide to involve the tele-EMS physician. The prompt availability of the tele-EMS physician facilitates the reduction in or alleviation of the immediate danger to the patient by providing immediate guidance. The path to establishing adequate patient care is determined as soon as possible.

The aim of this study was to analyze the telemedical consultations performed during life-threatening EMS missions and examine how these emergency patients benefited from the involvement of a tele-EMS physician.

## Methods

This retrospective, single-center cohort study was initiated and conducted at the Aachen Institute for Rescue Management & Public Safety in collaboration with the Department of Anesthesiology, RWTH Aachen University, Germany.

All data were extracted from the digitalized records of tele-EMS missions conducted over the course of four years (01/2015 until 12/2018). Obvious mistakes in documentation were reduced by plausibility checks before the statistical analyses. Ethics approval for the study was obtained from the medical faculty’s ethics committee (RWTH Aachen University, Pauwelsstraße 30, 52,074 Aachen, Germany; Number of approval: 109/15). The ethics committee, the university research board (Center for Translational and Clinical Research), and data protection officers granted the analysis of the data for quality assurance purposes and waived the requirement of informed consent from patients. All analysis were performed in accordance with relevant named guidelines and regulations; no experiments on humans were performed.

### Technical implementation of tele-EMS system

The teleconsultation center and ambulances equipped to enable teleconsultations form the two main pillars of the telemedicine system. Every ambulance is equipped with a mobile communication unit attached to the defibrillator monitor (corpuls C^3^; GS Elektromedizinische Geräte G Stemple, Kaufering, Germany) to enable real-time data transmission of the following components: audio; data on vital signs, including heart rate, blood pressure, oxygen saturation, 6 or 12-lead ECG; photos from the site taken with a smartphone; video streaming from a camera inside the ambulance controlled by the tele-EMS physician; and GPS coordinates of the ambulance to enable location tracking. Details regarding the technical setup of the system can be explored in earlier publications on the system’s technical performance^2,4,14^.

### Recorded data

All transmitted data is received by the teleconsultation center and displayed to the tele-EMS physician by software specifically developed for this purpose (Telemedical documentation; umlaut telehealthcare, Aachen, Germany). All obtained values, diagnostic measures and therapeutic interventions are documented throughout the teleconsultation, as in standardized emergency protocols, and stored electronically. These include all standard vital signs and diagnostic examinations performed according to the ABCDE (Airway, Breathing, Circulation, Disability, Environment) approach as well as the SAMPLE (symptoms, allergies, medication, past medical history, last oral intake, events prior to incident) approach. Furthermore, the tele-EMS physician documents all therapeutic interventions, such as the delegation of drug administration, and tracks the evolution of patients’ vital signs.

### Qualification of staff

All active tele-EMS physicians are board-certified anesthesiologists and additionally work as onsite EMS physicians. After they have completed at least 500 onsite emergency operations they are invited to take a specific assessment, on which they must achieve a passing score of 90% to pass^15^. After successfully passing the assessment, they undergo one week of supervised training in the teleconsultation center before taking over independent responsibility for missions. Paramedics participate in a one-day technical instruction course to learn how to use the equipment and a consultation training course to learn how to follow the structured sequence of a mission.

### Eligibility criteria

**Figure 1:**
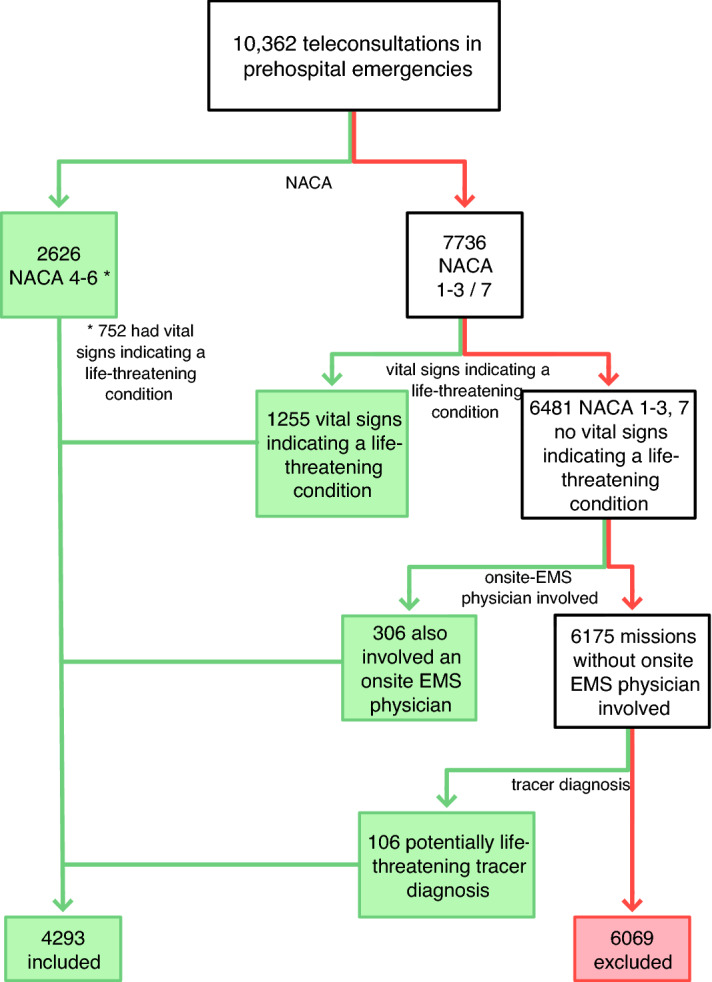
Flow chart of the inclusion of missions from all tele-EMS missions performed from 01/2015–12/2018; red arrow = exclusion, green arrow = inclusion.

All emergency missions were classified as life-threatening when at least one of the following eligibility criteria was met, as shown in Fig. 1:

- Documented patient severity scores of 4/5/6. The score was adapted from the National Advisory Committee for Aeronautics (NACA) and is commonly used to assess medical emergencies in several European countries (NACA 1 = minor disturbance, 2 = moderate disturbance, no measures necessary, 3 = severe but not life-threatening disorder, 4 = potentially life-threatening, 5 = acute risk of death, 6 = cardiac arrest, 7 = death).
- Life-threatening vital signs (airway obstruction, oxygen saturation (SpO_2_) < 90%, respiratory rate (RR) > 20 or < 6 per minute, systolic blood pressure (BP) < 90 or > 200 mmHg, heart rate > 140 or < 50 beats per minute, Glasgow Coma Scale (GCS) < 9, blood glucose level (BG) < 60 or > 500 mg/dl, body temperature (Temp) > 40 °C).
- An onsite EMS physician was involved in the mission (either because they were called to the scene by the tele-EMS physician, or because the consultation was only intended to bridge the time to arrival while the onsite EMS physician was en route) independent from the NACA score and vital signs.
- Major life-threatening tracer diagnoses (such as ST-elevation myocardial infarction (STEMI), new left bundle branch block (LBBB), acute coronary syndrome (ACS), cardiac decompensation, lung edema, acute dyspnea, coma, stroke, seizure, and acute suicidality).

Missions during which an onsite EMS physician was called to a stable patient with no abnormal vital signs because the paramedics could not establish intravenous access were excluded from the analysis.

When applying the eligibility criteria, vital signs were clustered according to the ABCDE model and the NACA score, and primary diagnoses were assessed based on the documentation from the tele-EMS physicians.

### Primary and secondary outcomes

The primary outcome parameter was the proportion of successfully improved life-threatening vital signs. For this purpose, the values of the vital signs were compared at the start and end of the tele-EMS consultation. The cutoffs for vital signs were those mentioned in the eligibility criteria. Furthermore, the need to call for an onsite EMS physician was measured, and tracer diagnoses of life-threatening conditions were analyzed. The secondary outcomes were administered drug doses and the general quality of the documentation of the missions. They were represented by the frequency of omitted values for analyzed vital signs.

### Statistical methods

Descriptive analysis for this study was conducted using Microsoft^®^ Excel for Office365 (Microsoft Corporation, Washington, USA). Statistical significance was tested using Fisher’s exact test and unpaired t-tests. The threshold for significance was a p-value less than 0.05. To minimize selection bias, diversified eligibility criteria were evaluated with independent parameters indicating a possible threat to life, including the NACA score, vital signs, and involvement of an onsite EMS physician.

Missing values naturally occur in analysis of medical records. There were no means to complement missing values of prehospital missions retrospectively. The proportions of missing values of vital signs were analyzed and are addressed later.

### Ethics approval and consent to participate

Ethics approval for the study was obtained from the medical faculty’s ethics committee at RWTH Aachen University, Pauwelsstraße 30, 52074 Aachen, Germany (Number of approval: 109/15). The ethics committee, the university research board (Center for Translational and Clinical Research), and data protection officers granted the analysis of the data for quality assurance purposes and waived the requirement of informed consent from patients.

## Results

During the study period, a total of 10,362 tele-EMS physician missions were conducted; 4,293 (41.4%) involved potentially life-threatening cases according to the inclusion criteria. The main characteristics of patients in critical conditions were circulatory problems, followed by respiratory problems. See Table 1 for details. A total of 3441 (80.15%) of the 4293 life-threatening missions were completed without an EMS physician at the scene. The onsite EMS physician was involved in 852 (19.85%) out of 4293 missions. In 441 (10.27%) of these missions, the EMS physician was secondarily notified by the tele-EMS physician or the ambulance at the scene (in the following referred to as a 'secondary alarm'). When the onsite EMS physician was primarily notified at the same time as the ambulance and already en route, which occurred in 411 cases (9.58%), teleconsultation was conducted either to bridge the interval until the arrival of the onsite EMS physician or to gain a second opinion.

**Table 1:** Mission data, demographics, and number of life-threatening vital signs.

**Mission data and demographics**			
Overall teleconsultations (n)			10,362
Missions involving life-threatening cases (n)			4293
Age (years; mean ± sd)			66 ± 21
**Sex**		n	%
Male		1628	37.92
Female		1742	40.57
Missing data		923	21.5
Number of life-threatening vital signs*			
*Airway*		Not open	6
*Breathing*	SpO_2_ (%)	< 90	346
	RR (/min)	> 20	215
	RR (/min)	< 6	1
*Circulation*	BP (mmHg)	< 90	203
	BP (mmHg)	> 200	769
	HR (bpm)	< 50	145
	HR (bpm)	> 140	313
*Disability*	GCS (3–15)	< 9	88
	BG (mg/dl)	< 60	93
	BG (mg/dl)	> 500	19
*Environment*	Temp (°C)	> 40	36
Total number of critical vital signs			2234

Mission data and demographics (upper table); Breakdown of the numbers of life-threatening vital signs (lower table).

*Sd* standard deviation, *SpO*_*2*_ oxygen saturation, *RR* respiratory rate, *BP* systolic blood pressure, *HR* heart rate, *GCS* Glasgow Coma Scale, *BG* blood glucose, *Temp* temperature.

*SI unit depending on vital sign.

### Life-threatening vital signs

Out of 2007 missions with 2,234 life-threatening vital signs (227 patients showed more than one critical vital sign), a total of 1,465 (65.6%) improved during the teleconsultation. The improvement of initially problematic vital signs is shown in Table 2. Hemodynamic problems were the most frequent cause for consultation regarding critically ill patients (n = 1430). All hemodynamic vital signs (tachy- and bradycardia as well as hypo- and hypertension) significantly improved during the teleconsultation, and respiratory parameters (SpO2, RR) were all treated, providing significant benefits to the patients. Reduced consciousness was significantly improved in cases of hypoglycemia. In contrast hyperglycemia and fever did not significantly improve.

**Table 2:** Cohorts in missions involving patients with initially life-threatening vital signs.

Life-threatening vital signs (n = 2007)			Vital signs at start of tele-consultation (*)	Vital signs at end of tele-consultation (*)	Significant improvement in vital signs	Cases at start of tele-consultation	Cases at end of tele-consultation	Significant reduction in cases
Vital signs defined as life-threatening			Mean + sd	Mean + sd		n	n	p-value
*Airway*	not open					6	0	*p* = *0.0311*
*Breathing*	SpO_2_ (%)	< 90	82.66 ± 10.18	93.11 ± 7.57	*p* < *0.0001*	346	75	*p* < *0.0001*
	RR (/min)	> 20	26.82 ± 5.05	23.66 ± 5.77	*p* < *0.0001*	215	145	*p* < *0.0001*
	RR (/min)	< 6	6	n.a		1	0	
*Circulation*	BP (mmHg)	< 90	79 ± 7.98	104 ± 21.27	*p* < *0.0001*	203	56	*p* < *0.0001*
	BP (mmHg)	> 200	217 ± 13.12	182 ± 23.45	*p* < *0.0001*	769	170	*p* < *0.0001*
	HR (bpm)	< 50	41.66 ± 6.13	53.77 ± 21.31	*p* < *0.0001*	145	81	*p* < *0.0001*
	HR (bpm)	> 140	163.43 ± 22.58	136.82 ± 30.5	*p* < *0.0001*	313	144	*p* < *0.0001*
*Disability*	GCS (3–15)	< 9	6.26 ± 1.76	10.11 ± 3.77	*p* < *0.0001*	88	27	*p* < *0.0001*
	BG (mg/dl)	< 60	41.27 ± 11.35	87.09 ± 36.21	*p* < *0.0001*	93	20	*p* < *0.0001*
	BG (mg/dl)	> 500	552.9 ± 33.85	548.47 ± 35.34	p = 0.6955	19	18	p = 1
*Environment*	Temp (°C)	> 40	40.61 ± 0.46	40.52 ± 0.52	p = 0.4393	36	33	p = 0.8083

The left box shows the means and standard deviations for the vital signs in initially critically ill patients before and after teleconsultation. The right box shows the number of cases with initially life-threatening vital signs at the start and end of the teleconsultation.

*Sd* standard deviation, *SpO*_*2*_ oxygen saturation, *RR* respiratory rate, *BP* blood pressure, *HR* heart rate, *GCS* Glasgow Coma Scale, *BG* blood glucose, *Temp* temperature.

*Unit depending on vital sign, n.a. = data was not available. *Italicized* p-values are significant.

The most frequent diagnoses during teleconsultation were stroke, with 890 (20.7%) cases, followed by acute coronary syndrome without STEMI, with 509 (11.9%) cases, and cardiac arrhythmias, with 296 (6.9%) cases.

### Need for onsite EMS physicians according to tracer diagnoses

**Figure 2:**
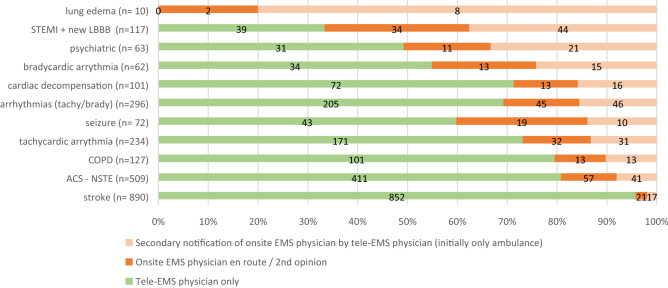
Distribution of tele- and onsite EMS physicians according to tracer diagnoses (brady- and tachycardic arrhythmias are subgroups of arrhythmias).

In 852 (19.85%) of all 4293 tele-EMS missions involving patients with potentially life-threatening conditions, an onsite EMS physician was involved at some point. Figure 2 shows the diagnoses made during the missions in which an onsite EMS physician was needed (orange units) in addition to the tele-EMS physician (green units) and differentiates whether the tele-EMS physician initiated the secondary alarm or the on-site physician was involved from the beginning. When there was a stroke, a physician was needed on the scene in 38 (4.2%) out of 890 cases because of impaired consciousness, while 852 stroke cases were handled by the tele-EMS physician alone. In contrast, all missions with severe pulmonary edema needed an onsite EMS physician to be present (100%). However, in eight of those ten cases (80%) the on-site physician was notified by the tele-EMS physician. The conventional EMS physician was further needed in 91 of 296 cardiac arrhythmia cases (30.74%). Differentiating between tachy- and bradycardic rhythms, bradycardia required the presence of an onsite EMS physician more often (18 out of 34; 52.94%) than tachycardia (63 out of 243; 25.92%).

### Drug administration delegated by tele-EMS physician

**Figure 3:**
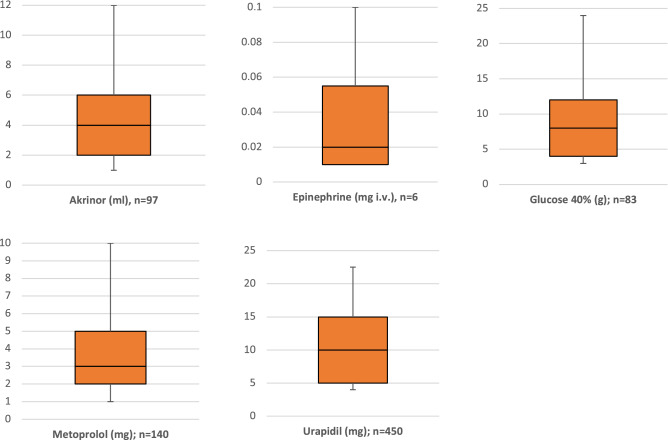
Drugs administered with the average cumulative dose per mission. Akrinor is a combination product containing both Cafedrin and Theodrinaline (200/10 mg/ml); ml = milliliter, mg = miligram, g = gram.

Urapidil was the most commonly recorded pharmaceutical administered during teleconsultations. It was administered at an average dose of 10 mg during 450 missions. Figure 3 depicts the most common drugs administered by the paramedics based on the guidance provided by the tele-EMS physician and their average dosages. In addition to the presented intravenous administration of epinephrine, this drug was also administered intramuscularly (IM) in five cases of severe anaphylaxis. In all five cases, the IM dose was 0.5 mg in accordance with the local SOPs and European Resuscitation Council (ERC) guidelines.

### Documentation quality

Of the total of 4293 missions the most common missing values were for temperature in 2,123 (49.5%) cases, respiratory rate in 2,101 (48.9%) cases, and blood glucose levels in 1,265 (29.5%) cases. In contrast, SpO2, BP and HR were well documented in greater than 95% of the tele-EMS physicians' missions, and GCS scores were documented in 92% (Table 3).

**Table 3:** Overall documentation quality of vital signs.

Overall documentation quality of vital signs (n = 4293)		
	Absolute number of missing values (n)	Relative portion of missing values (%)
Airway	131	3.05
SpO_2_	177	4.12
RR	2101	48.94
BP	180	4.19
HR	196	4.56
GCS	306	7.12
BG	1265	29.46
Temp	2123	49.45

Missing values are presented in absolute and relative numbers.

*SpO*_*2*_ oxygen saturation, *RR* respiratory rate, *BP* blood pressure, *HR* heart rate, *GCS* Glasgow Coma Scale, *BG* blood glucose, *Temp* temperature.

## Discussion

In this study, consultations with a tele-EMS physician during EMS missions involving patients in life-threatening conditions were analyzed. During the four years analyzed, a high proportion, 41.4%, of the missions were classified as involving patients in potentially life-threatening conditions. The primary conditions were respiratory and circulatory problems, which were resolved or at least significantly improved without an EMS physician on scene in 80.15% of the cases. A thorough evaluation of this type of telemedical emergency care involving life-threatening missions has not yet been reported in the literature.

### Improvements in vital signs indicating a life-threatening condition

In the prehospital phase, approximately two-thirds of the vital signs indicating a life-threatening condition could be remedied during the teleconsultation, usually without using invasive measures. The significant increase in oxygen saturation to above the critical level of 90% and the decrease in elevated respiration rates were mainly due to the provision of support with basic procedures as adequately opening the airway, elevated positioning of the patient and administering sufficient oxygen^16^. In contrast, circulatory problems, depending on their origin, need to be addressed with diverse interventions. The administration of stabilizing drugs, such as catecholamines and antiarrhythmics, or the administration of intravenous fluids can be effective. The majority of vital signs suggesting an immediate threat to the life of the patient, could be significantly improved during the teleconsultation without the need for invasive measures, such as intubation, artificial ventilation or electrical cardioversion. These findings are in line with the existing literature, which suggests that invasive measures are rarely performed in the prehospital phase of emergency cases^17^.

In this study, one-third of vital signs did not significantly improve during the teleconsultation. These cases mainly involved patients with elevated blood glucose levels and elevated body temperatures. While evidence-based methods for prehospital cooling have been widely reported, recommendations for the management of a patient’s temperature in the prehospital period have mainly been derived from resuscitation research^18,19^. There are no overall recommendations for the prehospital treatment of fever; most recommendations apply to the intensive care setting. Given the short prehospital timeframe, it is understandable that body temperature, which was not the focus of treatment, was not significantly improved.

With reference to patients with severe hyperglycemia, previous studies reported that greater than 90% needed to be hospitalized, mainly due to the need for controlled insulin treatment and frequent blood gas analyses^20^. Although prehospital point-of-care testing is feasible and available in some regions, these measures are not available in the prehospital care setting in the study region^21,22^. Recommendations for prehospital treatment mainly refer to the administration of intravenous fluids.

### Telemedical management of tracer diagnoses

Over the years, the indication index used to determine whether to notify the onsite EMS physician in the city of Aachen has been adapted to the 24/7 availability of telemedical support. In particular, many cases of stroke (without the loss of consciousness) and acute coronary syndrome without ST-elevation (NST-ACS), can be handled by the paramedics in consultation with the tele-EMS physician in many cases and usually do not require invasive measures. Data in this study strongly supported these findings, even when the cases were classified as potentially life-threatening, and the patients had impaired vital signs at the beginning of the teleconsultation.

The indication index also stipulates that an onsite EMS physician is always notified for patients with STEMI, due to the increased risk of malignant arrhythmias or cardiac arrest^23,24^. Despite this stipulation, there were 39 recorded cases of STEMI or new LBBB that were solely handled by the paramedics in consultation with the tele-EMS physician. Reasons for the absence of a secondary alert in those 39 cases include the strategic advantage of the geographical location of the ambulance, which may have had a shorter time to arrival at cardiac catheter centers than the estimated time to arrival of the onsite EMS physician at the scene, and the lack of availability of an onsite EMS physician.

In addition, an onsite EMS physician is also needed for all cases of lung edema, as severe lung edema often requires non-invasive ventilation with the application of continuous positive airway pressure (CPAP) or even invasive ventilation with endotracheal intubation. To perform these interventions, an onsite EMS physician is needed in the German EMS system^25,26^. Therefore, the fact that only 10 cases of lung edema were treated without an onsite EMS physician present reflects the recognition of this tracer diagnosis by the dispatcher during the call and the subsequent decision to immediately send an onsite EMS physician to the scene. Therefore, the tele-EMS physicians are rarely involved in these cases.

### Delegation of drug administration

When cardiac problems were detected, the tele-EMS physician delegated the administration of the appropriate drugs by the paramedics. These drugs included catecholamines in addition to drugs that are used to lower the patient’s blood pressure and heart rate. In many cases, this not only remedied the immediate critical condition but also minimized the therapy-free interval resulting from the uncertainty of the paramedic or the lack of an onsite EMS physician. For the pharmaceutical management of an acute symptomatic hypertensive emergency (defined as a systolic blood pressure greater than 180 mmHg or a diastolic blood pressure greater than 110 mmHg), the guidelines recommend reducing the arterial pressure by > 25% in the first hour^27^. The national German EMS recommendations suggest the administration of an initial dose of 5 mg of urapidil during prehospital management^28^.

Additionally, the intramuscular doses of epinephrine administered by the paramedics on the recommendation of the tele-EMS physician in cases of acute anaphylaxis were equivalent to the ERC recommendations and S2 guidelines in all cases^29^.

To control tachycardic atrial fibrillation, betablockers are the first-line recommendation in the national and international guidelines^30^. Metoprolol is the most commonly available drug in the prehospital setting and the administration of 2,5–10 mg is recommended to reduce the heart rate. In this study, the average cumulative dose was 3 mg. This reflects a relatively cautious strategy, likely due to the well-known negative inotropic side effects of betablockers, especially in patients with reduced ejection fraction of the left ventricle^31^.

Akrinor, which contains the catecholamines Cafedrin/Theodrinaline 200/10 mg/ml, had administered doses corresponding to those commonly used in clinical practice during anesthesia, as no specific prehospital recommendation is available^32^.

Overall, the doses recommended by the tele-EMS physicians were adequate for prehospital use. The earlier administration of drugs leads to a higher quality of patient care. The evaluation of the administration of pain medication was not a focus of this study and has been reported previously^6–8^.

### Documentation quality

In the setting of prehospital emergency care, the quality of documentation is important for several reasons: achieving an adequate handoff to the clinical care provider, legal investigations, trauma or CPR registries, research, and quality improvement initiatives^33^. Previous research in this field has shown improved documentation quality with regard to medical history and vital signs when telemedical emergency care is used than when conventional EMS care is provided^6,34^. In the records analyzed in this study, an overall high level of complete documentation was found, although there were several individual parameters that need more consistent documentation (49% availability for data on the respiration rate, temperature and blood glucose level). In past studies, the percent of cases with adequate RR documentation varied from 10–40%, and a recent systematic review on data quality in EMS reported that there were deficiencies in the completeness and accuracy of the reporting^35–37^. Therefore, the documentation of tele-EMS missions appears to be of relatively higher quality in terms of the completeness of the data.

### Feasibility of teleconsultation

This study shows that voluntary teleconsultation is often used by paramedics regarding critically ill patients when there is no onsite EMS physician and that the consultations were feasible to conduct. It contradicts the argument that there is no time for a teleconsultation regarding critically ill patients before the onsite EMS physician arrives. Anamnesis and, above all, diagnostic measures are the focus; nevertheless, there seems to be enough time for a teleconsultation after those initial measures, and teleconsultation can be integrated into the structured handling of the mission. Even if paramedics have been trained to perform multiple procedures on their own, there are several scenarios in which teleconsultation remains beneficial. As existing SOPs are usually selected based on a specific suspected diagnosis, uncertainty regarding the tracer diagnosis leaves the paramedic with the problem of deciding which SOP to follow. Furthermore, a medical problem may persist after working through an SOP or the patients’ problem may not match an SOP. In these cases, the paramedics may reach their limits of therapeutic options and can request additional assistance from the tele-EMS physician.

The prehospital application of telemedicine is rapidly expanding. The challenges of an increasing number of emergency calls; shortages of EMS physicians, especially in rural areas, which leads to prolonged response times; and outdated communication systems, require innovative approaches to the delivery of high-quality patient care. This study showed that prehospital telemedicine is also beneficial for patients in life-threatening conditions, even if it is not the primary goal to manage life-threatening emergencies without onsite EMS physicians. However, dispatchers sometimes cannot identify cases as life-threatening based on the call. In such cases, the tele-EMS physician can safely support the paramedics, allowing them to provide early and high-quality care during missions involving patients in life-threatening conditions. Further research in the field will be needed using mixed-methods to explore the different perspectives on tele-EMS, as well as focusing on patient outcomes after the prehospital phase^38^.

### Limitations of the study

This study specifically analyzed missions involving consultations with the tele-EMS physician regarding patients in life-threatening situations. It did not investigate missions involving patients in life-threatening conditions treated solely by the onsite EMS physicians and does not reflect the overall number of critically ill patients treated by the EMS system. Furthermore, there is the possibility that an ambulance team resolved a critical situation without consulting with a tele-EMS physician and without the help of the onsite EMS physician. The holistic tele-EMS system in the city of Aachen, where this analysis was conducted, is one of the most highly developed systems for routine prehospital emergency care worldwide. This impairs the transferability of the conclusions to other telemedicine systems. The authors recognize the real-time transmission of vital signs as one crucial aspect of telemedical applications in the context of prehospital emergency medicine.

## Conclusion

The consultation with a tele-EMS physician during missions involving patients in life-threatening conditions can significantly improve patients' vital parameters. Many cases could be addressed with the help of the tele-EMS physician and did not require invasive interventions by an onsite EMS physician. The delegation of the administration of drugs by a tele-EMS physician can often resolve a life-threatening condition. Teleconsultation not only helped resolve critical conditions but also minimized the therapy-free interval for the patients. Diagnoses, such as STEMI, cardiac pulmonary edema, or malignant dysrhythmias, necessitate the presence of onsite EMS physicians. Overall, even during missions involving patients with life-threatening conditions, teleconsultation was feasible and frequently conducted by the paramedics.

## Data Availability

The datasets analyzed during the current study are not publicly available due to the fact that the datasets are municipal property and cannot be published online under open access agreements. However, the datasets are available on reasonable request and with the permission of the municipal authorities.

## Abbreviations

ACS: Acute coronary syndrome
BG: Blood glucose level
BP: Blood pressure
EMS: Emergency medical service
ERC: European resuscitation council
GCS: Glasgow coma scale
HR: Heart rate
i.m.: Intramuscular
i.v.: Intravenous
LBBB: Left bundle branch block
NACA: National Advisory Committee for Aeronautics
RR: Respiration rate
SOPs: Standard operating procedures
SpO_2_: Oxygen saturation
STEMI: ST-elevation myocardial infarction
tele-EMS: Telemedical emergency medical service
Temp: Temperature
